# Cryoballoon ablation for atrial fibrillation: Effects on neuromodulation

**DOI:** 10.3389/fcvm.2022.958316

**Published:** 2022-07-28

**Authors:** Alvise Del Monte, Luigi Pannone, Antonio Bisignani, Thiago G. Osório, Saverio Iacopino, Gian-Battista Chierchia, Carlo de Asmundis

**Affiliations:** ^1^Heart Rhythm Management Centre, Postgraduate Program in Cardiac Electrophysiology and Pacing, Universitair Ziekenhuis Brussel - Vrije Universiteit Brussel, European Reference Networks Guard-Heart, Brussels, Belgium; ^2^Arrhythmology Department, Maria Cecilia Hospital, Cotignola, Italy

**Keywords:** cryoballoon ablation, atrial fibrillation, cardiac autonomic nervous system, autonomic denervation, ganglionated plexi, neuromodulation

## Abstract

Pulmonary vein isolation (PVI) represents the mainstay of atrial fibrillation (AF) ablation, and PVI with cryoballoon catheter (CB) ablation (CB-A) has proven to be as effective and safe as radiofrequency ablation (RF-A). Although AF is initiated by triggers arising from the pulmonary veins (PV) and non-PV foci, the intrinsic cardiac nervous system (ICNS) plays a significant role in the induction and maintenance of AF. The ICNS is an epicardial neural system composed of ganglionated plexi (GPs) and a complex network of interconnecting neurons. In the left atrium, the major GPs are located in proximity to the PV-left atrial junction. Vagal reactions have been described as markers of autonomic modulation during PVI with both RF-A and CB-A. The occurrence of neuromodulation during PVI with CB-A may be explained by both the anatomical relationship between the GPs and the PVs and the characteristics of the CB. Due to the CB/PV size mismatch, the CB creates a wide ablation area that extends from the PV ostium toward the antrum, possibly including the GPs. Although targeted GPs ablation, as a supplemental strategy to PVI, has been associated with a better AF outcome in patients undergoing RF-A, the additional clinical benefit of neuromodulation during PVI with CB-A remains a matter of debate. In this review, we provide an overview of the anatomy of the ICNS, the relationship between the ICNS and AF pathophysiology, and the current evidence on the clinical relevance of neuromodulation during PVI with CB-A.

## Introduction

Atrial fibrillation (AF) is known to be initiated by arrhythmogenic triggers originating from pulmonary vein (PV) and non-PV foci (1–3). According to the current guidelines, electrical isolation of the pulmonary veins (PVI) remains the cornerstone of AF ablation (4), and PVI with cryoballoon catheter (CB) ablation (CB-A) has been proven to be non-inferior to radiofrequency (RF) ablation (RF-A) with regard to safety and clinical outcomes (5).

Preclinical and clinical studies have shown that the intrinsic cardiac nervous system (ICNS) plays a crucial role in the induction and maintenance of AF through the regulation of different components of atrial cellular electrophysiology (6–9). The ICNS is an epicardial neural system composed of ganglionated plexi (GPs) and a complex network of interconnecting neurons that regulate cardiac electrical and mechanical functions on a beat-to-beat basis (10). In the atria, the major GPs are located in epicardial fat pads in close proximity to the PVs. Histological examination of the human heart has demonstrated that autonomic nerve density is mainly represented in the anterosuperior segments of both superior PVs and inferior segments of both inferior PVs, within 5 mm of the PV-left atrial (LA) junction, and in the epicardium (11).

The evidence of a vagal reflex, as a marker of GPs modification, during PVI with RF-A has posed the basis for the investigation of the role of neuromodulation on AF outcome. Several observational and few randomized studies have suggested that GPs ablation, as a supplemental strategy to PVI, might be associated with an additional clinical benefit in patients undergoing RF-A for both paroxysmal and persistent AF (12–14). Vagal reactions have also been reported during PVI with CB-A (15), especially during ablation of the left superior PV (16). The rationale behind neuromodulation during CB-A of AF is represented by both the close anatomical relationship between the GPs and the PVs, and the characteristics of the CB. Due to the size mismatch between the CB and the PV, the balloon is often in contact with atrial tissue distant from the PV orifice (17, 18), creating a large antral ablation area that can include the GPs (19). Although sometimes considered as part of the therapeutic effect (20), the clinical significance of GPs ablation in the context of CB-A for AF remains to be fully elucidated.

The aim of this review is to provide an overview of the anatomical basis, pathophysiological principles, and clinical significance of neuromodulation in the context of CB-A for AF.

## Anatomy of the cardiac autonomic nervous system: Relevant concepts for neuromodulation

The autonomic innervation of the heart relies on both sympathetic and parasympathetic fibers (21) and it can be anatomically divided into the extrinsic cardiac nervous system (ECNS) and the ICNS.

The ECNS consists of the nuclei in the brain stem, the ganglia along the cervical and thoracic segments of the spinal cord, and their axons that converge to the heart. Preganglionic sympathetic neurons arise from the spinal cord, synapse with the second sympathetic neurons in the paravertebral ganglia (mainly the cervical and stellate ganglia), and emit postganglionic axons that innervate the cardiomyocytes. Preganglionic parasympathetic neurons are located primarily in the dorsal vagus nerve and the nucleus ambiguus, and synapse with the second parasympathetic neurons, which lie in the epicardial clusters of autonomic ganglia, known as GPs (22, 23).

The ICNS is a complex epicardial neural network composed of the epicardial GPs, afferent and efferent nerve axons, and interconnecting neurons. GPs contain both sympathetic and parasympathetic elements, as well as multiple neuropeptides and neuromodulators, including calcitonin gene-related peptide, vasoactive intestinal polypeptide, substance P, and nitric oxide (24). The function of GPs is not only to modulate the autonomic interplay between the ECNS and the ICNS as “integration-centers” but also to independently regulate cardiac electrical and mechanical functions through the transduction of local signals (25). Epicardial ganglia in the human heart are present in both the atria and the ventricles and range in size from those containing a few neurons to large ganglia that measure up to 1 mm, containing over 200 neurons (26).

The anatomical localization of the atrial GPs represents a matter of scientific interest, especially for the identification of specific targets for arrhythmia treatment. The most commonly used classification of the atrial GPs in experimental and clinical studies is the one described by Armour et al. (26). The authors identified five major atrial GPs: (1) the superior right atrial GP, located on the posterosuperior surface of the right atrium (RA) adjacent to the junction of the superior vena cava (SVC) and the RA; (2) the superior left atrial GP, located on the posterosuperior surface of the LA between the PVs; (3) the posterior right atrial GP, located on the posterior surface of the RA, adjacent to the interatrial groove; (4) the posteromedial left atrial GP, located on the posteromedial surface of the LA; and (5) the posterolateral left atrial GP, located on the posterolateral surface of the LA base. The septal extensions of the posterior right atrial and posteromedial left atrial GPs form the interatrial septal GP.

**Figure 1:**
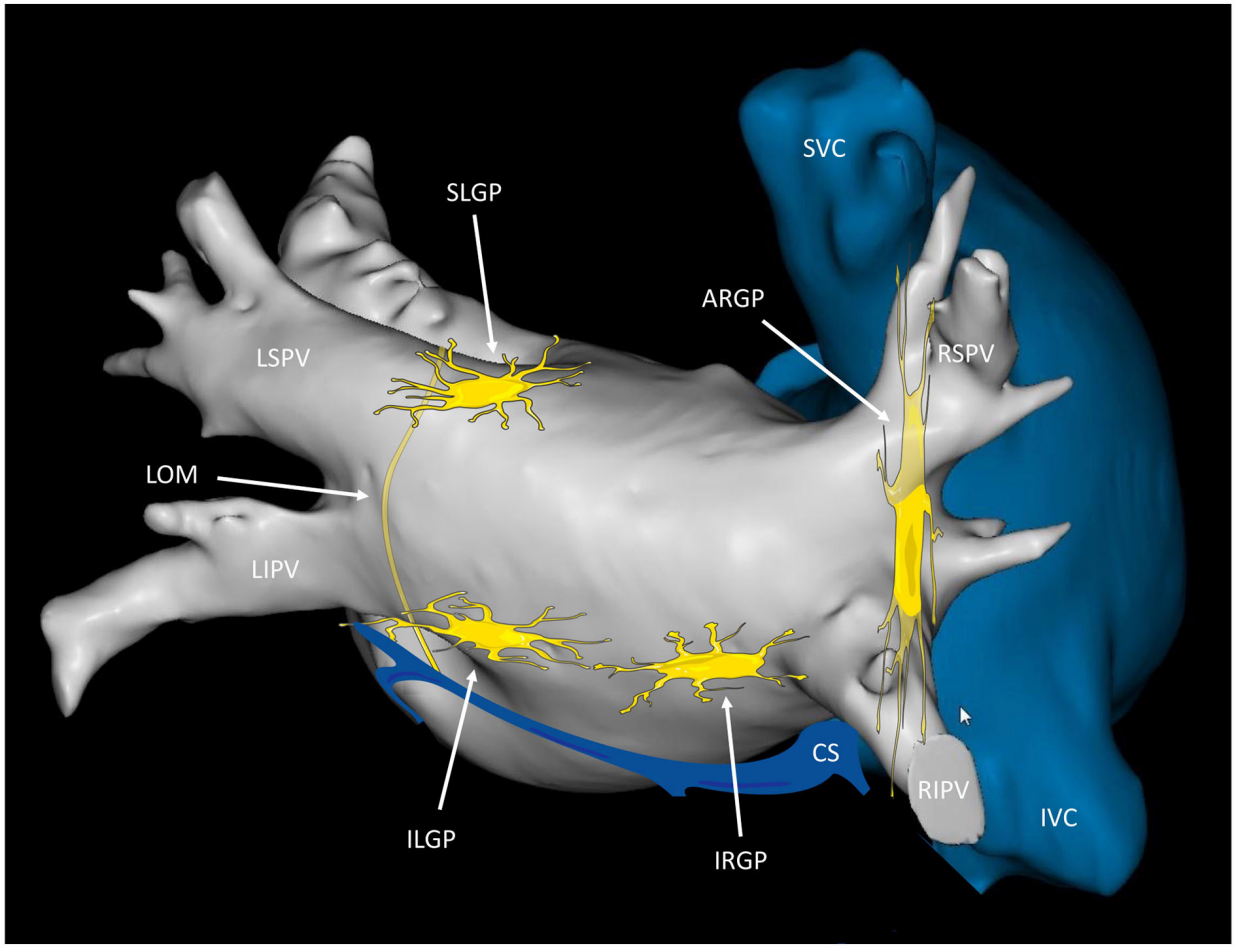
Anatomical localization of the major left atrial ganglionated plexi. Posterior view of the left and right atria displaying the presumed location of the major atrial ganglionated plexi (GPs) and the ligament of Marshall (LOM). The SLGP is located on the roof of the left atrium (LA), near the LSPV-LA junction; the ILGP is situated at the inferior aspect of the posterior wall of the LA; the ARGP is located anterior to the RSPV; the IRGP is situated at the inferior aspect of the LA. The ILGP and the IRGP are normally located 1–3 cm below the lower edge of the inferior pulmonary veins. SLGP, superior left GP; ILGP, inferior left GP; ARGP, anterior right GP; IRGP, inferior right GP; LSPV, left superior pulmonary vein; LIPV, left inferior pulmonary vein; RSPV, right superior pulmonary vein; RIPV, right inferior pulmonary vein; CS, coronary sinus; SVC, superior vena cava; IVC, inferior vena cava.

For easier communication between electrophysiologists, the GPs have been renamed according to their relationship with the PVs (27): (1) the superior left GP (SLGP), located on the roof of the LA, 1–2 cm medial to the left superior PV; (2) the anterior right GP (ARGP), located anterior to the right superior PV; (3) the inferior left GP (ILGP); and (4) the inferior right GP (IRGP), situated at the inferior aspect of the posterior wall of the LA, 1–3 cm below the inferior PVs (Figure 1).

The concept of GPs as a grouping of ganglia in different epicardial sites has been challenged by later studies on whole heart preparations, which demonstrated that the GPs are densely interconnected by thinner nerves and should be considered as distinctive ganglionated areas of a single continuous cardiac ganglionated nerve plexus, from which intrinsic nerves extend to distinct cardiac regions (28). Both the ganglia and their connections were defined as ganglionated subplexi (sGP). In the atrium, 5 sGPs were identified: (1) the ventral right atrial sGP, which covers the ventral superior RA, the ventral side of the root of the SVC, and the ventral inferior RA; (2) the ventral left atrial sGP, which covers the ventral superior LA; (3) the left dorsal sGP, which covers the region across the left coronary sulcus; (4) the middle dorsal sGP, which covers the dorsal superior LA and the region around the crux cordis; and (5) the dorsal right atrial sGP, which covers the dorsal superior RA, the dorsal side of the root of the SVC, and the region over the interatrial septum (29).

Although different classifications exist for the identification of atrial GPs, there is considerable anatomical overlap between them (Table 1).

**Table 1:** Anatomical classifications of epicardial ganglia.

**Classification**	**Anatomical location**					
	**SVC-RA**	**RSPV**	**RIPV-IAS**	**LSPV-LA roof**	**LIPV-LA PW**	**LOM**
Armour et al. (26)	SRAGP	SRAGP	PMLAGP	SLAGP	PLLGP	/
Po et al. (27)	/	ARGP	IRGP	SLGP	ILGP	LOM
Pauza et al. (29)	VRAsGP	DRAsGP	DRAsGP/MDsGP	MDsGP/LDsGP	MDsGP/LDsGP	LDsGP

*SVC, superior vena cava; RA, right atrium; RSPV, right superior pulmonary vein; RIPV, right inferior pulmonary vein; IAS, interatrial septum; LSPV, left superior pulmonary vein; LA, left atrium; LIPV, left inferior pulmonary vein; PW, posterior wall; LOM, ligament of Marshall; GP, ganglionated plexus; SRAGP, superior right atrial GP; PMLAGP, posteromedial left atrial GP; SLAGP, superior left atrial GP; PLLGP, posterolateral left atrial GP; ARGP, anterior right GP; IRGP, inferior right GP; SLGP, superior left GP; ILGP, inferior left GP; sGP, subganglionated plexus; VRAsGP, ventral right atrial sGP; DRAsGP, dorsal right atrial sGP; MDsGP, middle dorsal sGP; LDsGP, left dorsal sGP*.

The innervation of the sinus node (SN) and the atrioventricular node (AVN) is mediated by different GPs (30). The SN is primarily regulated by the ventral and dorsal right atrial sGPs (corresponding to the anterior right GPs), whereas the AVN is supplied by fibers originating from the left dorsal, middle dorsal, ventral right, and dorsal right sGPs, extending toward the interatrial septum (corresponding to the inferior right GP).

Another component of the ICNS is represented by the ligament of Marshall (LOM), which is a developmental vestige of the embryonic left SVC, described by John Marshall in 1850 (31). This structure is innervated by predominantly parasympathetic fibers, which originate from the left vagus, travel through the LOM, and innervate surrounding left atrial structures, including the PVs, left atrial appendage, coronary sinus, and posterior left atrial fat pad. The vagal effects mediated by the LOM in the left atrium provide the basis for its arrhythmogenic role in the genesis and maintenance of AF (32).

## Pathophysiology of atrial fibrillation: The role of the autonomic nervous system

Pathophysiology of AF results from a complex interplay between triggers (3), drivers (33), and substrate (34). In this context, the autonomic nervous system can modulate different components of atrial cellular electrophysiology inducing and sustaining AF (35). Animal studies demonstrated that all AF episodes are preceded by the activation of the ICNS and that its ablation can reduce atrial vulnerability, thus suggesting the importance of the ICNS in AF arrhythmogenesis (36, 37).

PVs are a known source of AF triggers due to the short action potential duration (APD) of PV cells, the APD gradient at the PVs-LA junction, and the short coupling interval for excitation (38). Therefore, excitation during repolarization, or early after depolarizations (EADs), is the result of the typical electrophysiological characteristics of PV cells. In canine PVs preparations, pacing-induced EADs were able to generate only rare single beats and never repetitive rhythms when exposed only to catecholamines. With combined exposition to catecholamines and acetylcholine, pacing-induced EADs were able to provoke rapid firing from PVs (39). This finding suggests a role of both sympathetic and parasympathetic effects in AF initiation. Sympathetic tone enhances the Ca2+ transient, characterized by an increase in calcium entry, storage, and release in the heart (40). Parasympathetic activity is mediated by the activation of the acetylcholine-activated potassium channel (I_K−Ach_), which decreases the APD (39). The final combined result promotes the development of late phase 3 EAD-induced triggered activity in the atria and the PVs (41).

Furthermore, parasympathetic stimulation induces a significant increase in the heterogeneity of the atrial effective refractory period (ERP) (42), which may allow multiple-circuit reentry by reducing minimum circuit size and allowing more circuits to be accommodated in the atria (34).

The autonomic nervous system may play a role in arrhythmogenesis also inducing atrial electrical remodeling, thus contributing to AF perpetuation. In animal models, rapid atrial pacing (RAP) simulating AF was able to induce electrical remodeling, characterized by a progressive reduction of ERP, progressive increase in ERP dispersion, and enhanced AF inducibility. This electrical remodeling induced by RAP could be prevented by GPs ablation or autonomic pharmacological blockade with atropine and propranolol (43). RAP could also induce autonomic remodeling, characterized by increased parasympathetic and sympathetic activity, which was able to further enhance electrical remodeling, therefore creating a vicious cycle causing AF perpetuation (“AF begets AF”) (44).

Thus, the elimination of adrenergic and cholinergic fibers within GPs might improve outcomes in patients with AF. The coexistence of adrenergic and cholinergic fibers in the GPs renders it nearly impossible to selectively ablate their adrenergic or cholinergic component. However, the anatomical distribution of the parasympathetic postganglionic neuronal bodies in the epicardial GPs makes parasympathetic reinnervation after ablation less likely (45).

## Autonomic denervation during cryoballoon catheter ablation for atrial fibrillation

### Principles of autonomic denervation with cryoballoon ablation

The presence of atrial GPs hyperactivity has been demonstrated before episodes of AF on animal models (36), but the exact extent of these “hyperactive” GPs is still unknown. Three techniques have been described to localize and target autonomic GPs: (1) the anatomic approach, which relies on GPs ablation at their presumed anatomical locations (14), (2) high-frequency stimulation (HFS; 20 Hz, 10–150 V, pulse width 1–10 ms for 2–5 s), which relies on the identification of HFS-evoked vagal responses (≥50% increase in mean R-R interval during AF) to differentiate GPs from atrial myocardium (27), and (3) electrogram analysis, to identify fragmented atrial potentials. Using fast Fourier transformation, Pachon et al. (45) defined compact and fibrillar atrial potentials during sinus rhythm and postulated that fragmented fibrillar atrial potentials were the markers of GPs insertion in the atrial tissue. Furthermore, the presence of at least four deflections in the signal at the ablation site has been demonstrated to be the best predictor of a vagal response during RF ablation (46).

HFS-induced vagal response has proven to be a specific but not sensitive method to target the GPs, probably due to the limited effect of the endocardial stimulation of the GPs which are located epicardially (27). Furthermore, despite its theoretical principle, RF ablation of GPs based on HFS has not been demonstrated to be superior to the anatomic approach (47). One postulated explanation is related to the wider ablation area in the anatomic approach compared to the HFS-based strategy, suggesting better results with more extensive regional ablation. Another possible explanation might be the relatively small anatomical variations of the main left atrial GPs, which lie close to the PV-left atrial junction.

These findings explain the rationale for the possible concomitant neuromodulation during CB-A for AF. PVI with CB-A, especially with the 28-mm CB catheter, is associated with a broad antral ablation area that extends from the PV ostium toward the LA (17, 18), thus increasing the possibility of concomitant and extended GPs ablation (19). Compared to RF-A, PVI with CB-A has been demonstrated to involve a wider surface of the ICNS (18). Common antral PVI with RF-A is typically characterized by concomitant transection of two of the four GPs (i.e., ARGP and SLGP) since the inferior GPs are typically distant from the inferior edge of the usual antral isolation area (27). CB-A of PVs with the 28-mm CB catheter, instead, can simultaneously affect all the major GPs, possibly including the hyperactive GPs, with a higher effect on the SLGP and ARGP and a smaller involvement of the ILGP, likely due to its anatomical variations (20). Indeed, a vagal response has been observed in 38.3% of the patients undergoing PVI with CB-A (48), while it has been reported only in 17% of patients during RF-A, mostly during ablation of the left PVs (49).

### Assessment of neuromodulation during cryoballoon ablation for atrial fibrillation

The effect of CB-A on the ICNS can be assessed both in the acute phase after PVI (i.e., intraoperatively or within 24 h after PVI) and in the longer term (after the first 24 h post-PVI) (Table 2).

**Table 2:** Neuromodulation assessment after cryoballoon ablation for atrial fibrillation.

**Method**	**Definition**	**Timing**	**Evidence**
Vagal reaction	Sinus bradycardia <40 bpm, asystole, atrioventricular block, or hypotension	Intraoperatively	40.7% of patients during balloon thawing and balloon deflation (19)
Extracardiac vagal stimulation (ECVS)	Quantification of vagal reaction (prolongation of the RR interval by >50%, and/or atrioventricular block) through vagus nerve stimulation by pulsed electric field before and after PVI	Intraoperatively	Varying degree of parasympathetic denervation in all patients undergoing PVI with CB-A for paroxysmal AF (mean pause duration 10,130.6 ± 3,280.0 ms pre-PVI vs. 1,687.5 ± 2,183.7 ms post-PVI, *p* < 0.001) (50)
Heart rate (HR) increase	Increase in HR after PVI compared to HR pre-PVI	24 h post-PVI or at 6 months	HR increased from 57.93 ± 9.06 bpm pre-PVI to 71.10 ± 12.75 bpm 24-h post-PVI and 62.59 ± 7.89 bpm 6 months post-PVI (*p* < 0.001) (51)
Handgrip test	Lowering in systolic blood pressure increase during handgrip test after PVI compared to pre-PVI	24 h post-PVI	30.50 ± 25.49 mmHg pre-PVI vs. 19.40 ± 22.40 mmHg 24 h post-PVI (*p* = 0.041) (51)
Heart rate variability (HRV)	HRV tested by Holter ECG for SDNN and TI before PVI and after 1 and 3 months post-PVI	1–3 months post-PVI	HRV decreased significantly immediately after PVI for both SDNN and TI until 1 month, gradually normalizing toward 3 months follow-up (52)

*PVI, pulmonary vein isolation; CB-A, cryoballoon ablation; AF, atrial fibrillation; SDNN, standard deviation of normal-to-normal intervals; TI, triangular index*.

In the acute phase, neuromodulation during CB-A may be demonstrated intraoperatively with the occurrence of a vagal reaction, defined as sinus bradycardia <40 bpm, asystole, atrioventricular block, or hypotension, which has been described during balloon thawing and balloon deflation in 40.7% of patients (19). Moreover, neuromodulation during PVI with CB-A can be assessed 24 h after PVI with an increase in heart rate (HR) and with a lowering in systolic blood pressure increase during the handgrip test (51). HR increase after PVI has been significantly associated with: age at ablation, baseline heart rate before CB-A, and nadir temperature in each right PV (53).

**Figure 2:**
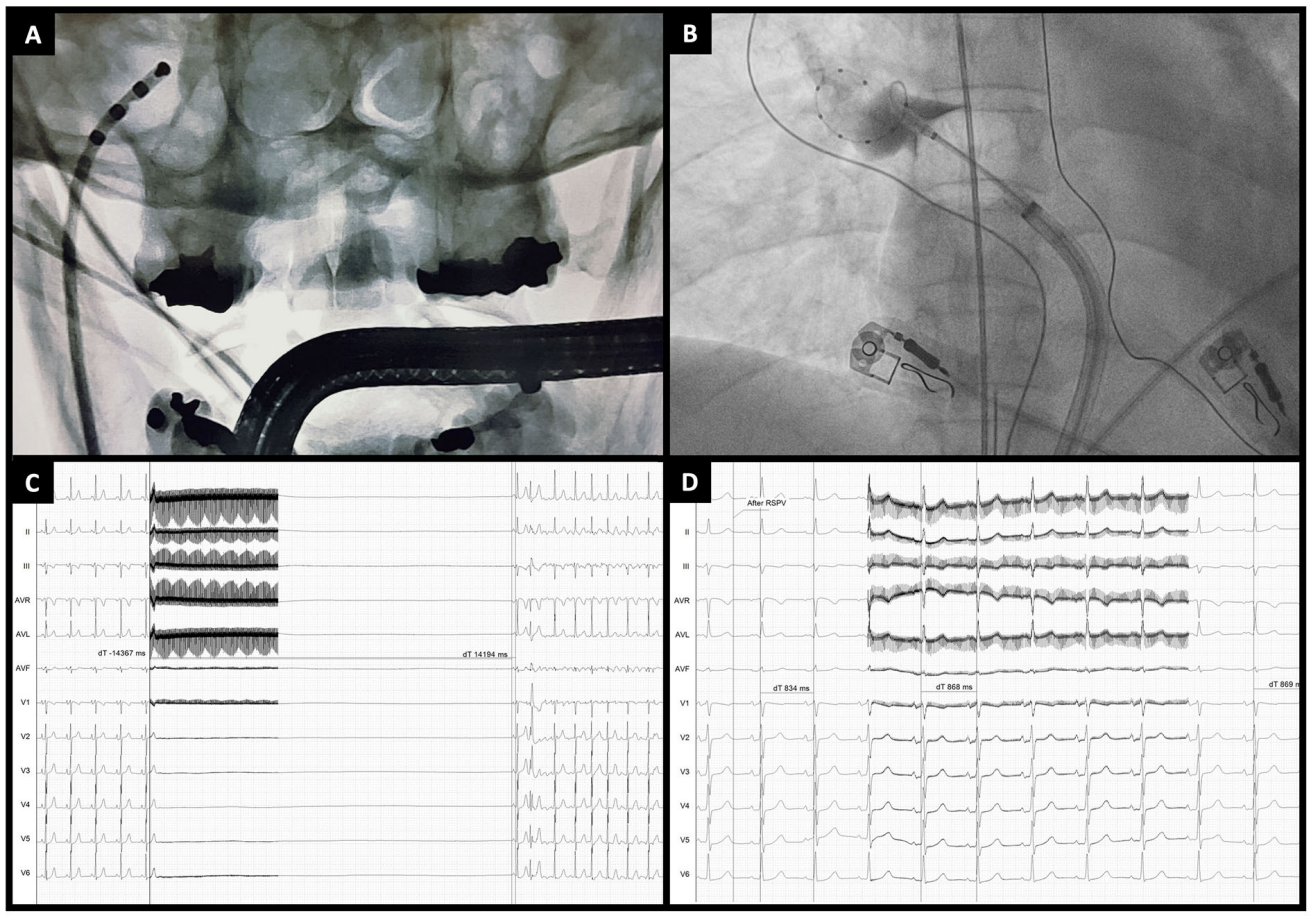
Extracardiac vagal stimulation during cryoballoon ablation. **(A)** Anteroposterior fluoroscopic view of a quadripolar catheter advanced through the right jugular vein to the jugular foramen for vagal stimulation. **(B)** Example of cryoballoon ablation of the right superior pulmonary vein (anteroposterior fluoroscopic view). **(C)** Extracardiac vagal stimulation before PVI showing a marked vagal response (asystole of around 14 s) followed by atrial fibrillation induction. **(D)** Extracardiac vagal stimulation at the end of cryoballoon ablation showing the absence of a vagal response (RR interval changes from 834 to 868 ms).

A method to reliably quantify the acute degree of vagal denervation is extracardiac vagal stimulation (ECVS) (54). In brief, a quadripolar catheter is advanced through the right femoral vein to the right internal jugular vein in the region of the jugular foramen, directed medially. Usually, in this place, there is proximity to the vagus nerve. ECVS is obtained by a pulsed electric field (pulse amplitude of 0.5–1 V/kg body weight up to 70 V, 50 ms pulse width, 50 Hz frequency, lasting 5 s) within the jugular vein to elicit a vagal response. The typical response is transitory sinus arrest or bradycardia, defined as prolongation of the RR interval by >50%, and/or atrioventricular block. ECVS is performed before and after PVI, and neuromodulation is confirmed if a reduction in the vagal response is achieved (Figure 2). In all patients with paroxysmal AF undergoing PVI with CB-A, ECVS demonstrated a varying degree of parasympathetic denervation, and ablation of the RSPV has been associated with the most significant acute reduction of parasympathetic tone (55). This response has proven to be more sensitive in quantifying vagal denervation than the increase in heart rate (50).

The longer-term (>24 h after PVI) effect of CB-A on vagal denervation has been described using HR and Heart Rate Variability (HRV), as surrogates for GPs modulation. Oswald et al. (52), who were the first to investigate the effect of CB-A on the ICNS, demonstrated that in the majority of patients CB-A was associated with significant changes in HRV during follow-up. Furthermore, in a cohort of 103 patients undergoing PVI with the 28-mm second-generation CB catheter, increased HR persisted in 37.9% of patients even at 12 months after PVI (56).

### Prognostic impact of neuromodulation with cryoballoon ablation for atrial fibrillation

Numerous studies investigated the role of neuromodulation associated with CB-A of PVs on AF outcome (Table 3).

**Table 3:** Summary of the principal studies investigating the role of neuromodulation with cryoballoon ablation on atrial fibrillation outcome.

**References**	***N*** **. of patients**	**Type of AF**	**Type of CB**	**Neuromodulation assessment**	**Follow-up**	**Effect on AF outcome**
Oswald et al. (52)	14	Paroxysmal	Arctic Front, 28 mm	VR^*^ during CB-A, HRV during follow-up	3 months	No association with AF recurrence
Yorgun et al. (19)	145	Paroxysmal (*n* = 117) and persistent (*n* = 28)	Arctic Front, 28 mm	VR^*^ during CB-A	17 (4–27) months	VR was more common in patients without AF recurrence (46.2 vs. 15.4%, *P* = 0.004); VR requiring atropine or temporary pacing was an independent predictor of AF-free survival (HR 0.064; 95% CI 0.008–0.48, *p* = 0.008)
Aytemir et al. (57)	306	Paroxysmal (*n* = 247) and persistent (*n* = 59)	Arctic Front, 28 mm (*n* = 197) and Arctic Front Advance, 28 mm (*n* = 109)	VR^*^ during CB-A	22 (13–34) months	VR was a predictor of AF-free survival (HR 0.550, 95% CI 0.331–0.915, *p* = 0.021)
Miyazaki et al. (56)	103	Paroxysmal	Arctic Front Advance, 28 mm	VR^*^ during CB-A, increased HR during follow-up	15.0 (12.0–18.0) months	No association with AF recurrence (*p* = 0.300 and *p* = 0.224, respectively)
Te et al. (16)	39	Paroxysmal	Arctic Front Advance, 28 mm	VR^*^ during CB-A	14 ± 6 months	Increased AF-free survival of patients with VR compared to patients without VR (log rank *p* = 0.006)
Guckel et al. (58)	250	Persistent	Arctic Front Advance, 28 mm	VR^*^ during CB-A	12 months	VR was an independent predictor of AF-free survival (HR 0.11; 95% CI 0.03–0.34; *p* < 0.01)
Maj et al. (53)	472	Paroxysmal (*n* = 459) and persistent (*n* = 13)	Arctic Front Advance Pro, 28 mm	HR increase >15 bpm after CB-A compared to baseline	27.7 ± 12.5 months	Patients with HR increase >15 bpm presented higher AF-free survival (83.1 vs. 66.3%, respectively; Log Rank *p* = 0.021)
Călburean et al. (59)	110; CB-A *n* = 71	Paroxysmal (*n* = 72) and persistent (*n* = 38)	Arctic Front Advance Pro, 28 mm	DC on ECG Holter after CB-A	15.4 (7.0–28.2) months	Higher DC was an independent predictor of AF recurrence (HR 1.68, 95% CI 1.35–1.82, *p* = 0.004)
Tang et al. (60)	346; CB-A *n* = 231	Paroxysmal	Arctic Front Advance, 23- or 28-mm	HR increase after CB-A measured with ICM	12 months	Patients without AF recurrence presented faster daytime (11 ± 11 vs. 8 ± 12 bpm, *p* = 0.001) and nighttime HR (8 ± 9 vs. 6 ± 8 bpm, *p* = 0.049) compared to patients with AF recurrence

*AF, atrial fibrillation; CB, cryoballoon, CB-A, cryoballoon ablation; VR, vagal reaction; HR, heart rate; HRV heart rate variability; HR, hazard ratio; CI, confidence interval; DC, deceleration capacity; ICM, implantable cardiac monitor*.

* *Sinus bradycardia (heart rate <40 bpm), asystole, complete atrioventricular block, or hypotension (systolic blood pressure <90 mm Hg, diastolic blood pressure <60 mm Hg or a sudden drop in blood pressure from baseline by at least 20 mm Hg)*.

In patients with persistent AF, the occurrence of an intraprocedural vagal reaction, defined as bradycardia <40 beats/min, asystole, or high-degree atrioventricular block, was an independent predictor of AF-free survival after PVI with CB-A (58). Comparable results were confirmed also in patients with paroxysmal AF undergoing CB-A (16, 57). In this population, the occurrence of vagal reactions during PVI was associated with an inferior rate of AF recurrence in the mid-term. Similarly, an intraprocedural vagal reaction requiring atropine administration or temporary pacing decreased the risk of AF recurrence (19).

Furthermore, high parasympathetic activity after RF-A and CB-A of PVs has been associated with AF recurrence. An HR increase ≥15 bpm after CB-A compared to pre-PVI could stratify patients with a higher AF-free survival at a 2-years follow-up (53). Similarly, Tang et al. (60) observed that daytime and nighttime heart rates were significantly higher in patients without AF recurrence, compared to patients with AF recurrence. Moreover, high deceleration capacity (DC) at ECG Holter monitoring could predict AF recurrence after PVI with CB-A (59). Interestingly, high parasympathetic tone, expressed by DC and other surrogate markers, could predict not only AF recurrence but also PV reconnection (61). Right-sided only PVs reconnection was associated with higher parasympathetic tone than left-sided only PVs reconnection. This might be explained by a suboptimal lesion created during CB-A, with associated incomplete PVI and preserved function of GPs in the region of reconnected PVs. In particular, suboptimal lesions on right-sided PVs are associated with higher persistence of parasympathetic tone because of the preserved function of the ARGP, which represents the final common parasympathetic pathway to the SN.

Despite these results, other authors failed to prove any positive clinical correlation between autonomic modulation and AF recurrence after CB-A. In one study analyzing 103 patients with paroxysmal AF undergoing PVI with the 28-mm second-generation CB catheter, neither intra-procedural vagal reactions nor HR increase could predict AF recurrence (56).

Direct comparative data on RF-A vs. CB-A of GPs on AF outcome is scarce; however, some evidence suggests that no significant difference exists between the two ablation strategies (60, 62).

Along with its beneficial effect on AF outcome, GPs ablation has also been associated with a potential proarrhythmic effect. With RF-A, GPs ablation added to PVI has been reported to carry a higher risk of ablation-induced left atrial tachycardias than PVI alone, mostly due to the creation of supplemental atrial lesions (27). Selective anatomic or HFS-mediated radiofrequency GPs ablation has been complicated by atrial macroreentry in 2–10% of patients (14). A notably increased proarrhythmic risk has been described in animal models with selective GPs ablation without PVI, likely secondary to a decreased atrial ERP and a hyper-reinnervation of both sympathetic and parasympathetic nerves after 8 weeks (63). However, with CB-A, neuromodulation is a concomitant effect obtained during PVI, and no additional applications are delivered to selectively target the GPs. Therefore, the proarrhythmic effect related to concomitant neuromodulation during CB-A is the one related to PVI with the cryoballoon (64, 65), which may cause an increased heterogeneity of refractoriness within the atria.

## Discussion and future perspectives

The significant role of the ICNS in the initiation and maintenance of AF is well-known, however conflicting results still exist regarding the association between autonomic modulation during PVI with CB-A and AF outcome. The explanation of this phenomenon may be multifactorial.

First, adequate patient selection might play a major role in the additional benefit of neuromodulation on AF recurrence. Although GPs ablation has demonstrated additional benefits when associated with PVI in paroxysmal AF, the results of the AFACT study (66) demonstrated that GPs ablation during thoracoscopic surgical ablation of advanced AF had no beneficial effect on AF recurrence. This evidence may suggest that the therapeutic advantage of GPs ablation is more evident at an earlier stage of AF when the role of the ICNS in AF initiation and perpetuation is more significant.

Second, the use in the literature of different markers of autonomic modulation (e.g., vagal reaction, HR, and HRV) to evaluate the impact of autonomic denervation on AF recurrence limits the comparison between studies for outcome analysis. Furthermore, vagal reactions, HR increase, and decreased HRV may be imprecise in reflecting complete vagal denervation. The adoption of a standardized approach, like the ECVS, to assess vagal denervation after PVI may allow more reliable data on the real incidence and clinical significance of complete vagal denervation with CB-A. However, future studies are needed to support this theory.

Third, a potential limitation of the effect of neuromodulation on AF outcome in the long-term is the possible occurrence of reinnervation post-PVI, as already reported after both surgical excision and RF-A of GPs (67, 68). Prior studies on CB-A with the first-generation CB showed that autonomic modulation expressed as increased HR and decreased HRV, recovered after 3–6 months post-PVI (52). More recent evidence on CB-A with the second-generation CB reported a persistent effect on neuromodulation up to 12 months post-PVI (56). This difference might be explained by the more extensive ablation lesion created by the second-generation CB, which may cause destruction not only of nerve axons but also of nerve cell bodies. Similar durable effects of neuromodulation have also been described with RF-A (69).

Finally, autonomic modulation with CB-A is a concomitant effect obtained during PVI. Despite the evidence of a wide ablation area created by the CB catheter, GPs ablation during PVI may be incomplete in some cases, thus reducing its beneficial effect or increasing the arrhythmic risk. Only recently, different mapping systems, like the dielectric imaging and navigation system (KODEX-EPD™; EPD Solutions, Philips, Eindhoven, The Netherlands), have been developed to allow the visualization of the multipolar mapping catheter used during CB-A to facilitate the procedure (70). Future research in this field may focus on the implementation of those mapping systems (e.g., refined electrogram analysis) to precisely target the GPs and achieve complete GPs ablation during PVI with CB-A or perform selective neuromodulation with the CB catheter.

Besides RF-A and CB-A, new energy sources are available for the treatment of AF (71). Among them, pulsed-field ablation (PFA) is emerging as a promising non-thermal ablation modality that creates lesions through irreversible electroporation. One of the main advantages of PFA is its theoretical superior safety profile related to tissue selectivity for cardiac tissue, with no or minimal effects on adjacent structures (72). Animal studies demonstrated the preservation of phrenic nerve and esophageal tissue despite exposure to clinical PFA energies (73, 74). However, currently, no clinical data is available on the effect of PFA on the ICNS, and future studies are needed to determine its role in neuromodulation during AF ablation.

## Conclusion

Due to the anatomical proximity of the main left atrial GPs with the PVs and the characteristics of the CB, neuromodulation is a common concomitant effect obtained during PVI with CB-A. Although some evidence supports this theory, the real additional beneficial effect of neuromodulation during CB-A on AF outcome has still not been completely clarified.

## Author contributions

ADM and LP searched and analyzed the literature. ADM wrote the manuscript. LP, AB, and GBC reviewed and edited the manuscript. All authors have read and approved the final manuscript.

## Conflict of interest

Author AB is consultant for Biotronik. Author GBC received compensation for teaching purposes and proctoring from Medtronic, Abbott, Biotronik, Boston Scientific, and Acutus Medical. Author CdA receives research grants on behalf of the center from Biotronik, Medtronic, Abbott, LivaNova, Boston Scientific, AtriCure, Philips, Acutus Medical, and received compensation for teaching purposes and proctoring from Medtronic, Abbott, Biotronik, LivaNova, Boston Scientific, AtriCure, Acutus Medical, and Daiichi Sankyo. The remaining authors declare that the research was conducted in the absence of any commercial or financial relationships that could be construed as a potential conflict of interest.

## Publisher's note

All claims expressed in this article are solely those of the authors and do not necessarily represent those of their affiliated organizations, or those of the publisher, the editors and the reviewers. Any product that may be evaluated in this article, or claim that may be made by its manufacturer, is not guaranteed or endorsed by the publisher.
